# Imaging-Based Pre-Operative Differentiation of Ovarian Tumours—A Retrospective Cross-Sectional Study

**DOI:** 10.3390/diagnostics15202560

**Published:** 2025-10-11

**Authors:** Assel Kabibulatova, Mehzabin Kazi, Peter Berglund, Malin Båtsman, Ulrika Ottander, Sara N. Strandberg

**Affiliations:** 1 Scientific Research Institute of Radiology Named After ZH.H. Khamzabayev, Astana Medical University, Astana 010000, Kazakhstan; 2 Department of Diagnostics and Intervention, Diagnostic Radiology, Umeå University, SE-90187 Umeå, Sweden; 3 Department of Medical Biosciences, Pathology, Umeå University, SE-90187 Umeå, Sweden; 4 Department of Clinical Sciences, Obstetrics and Gynecology, Umeå University, SE-90187 Umeå, Sweden

**Keywords:** ovarian neoplasms, neoplasm staging, magnetic resonance imaging, X-ray computed tomography

## Abstract

**Objectives**: This study aimed to investigate the diagnostic performance of imaging-based biomarkers from computed tomography (CT) and magnetic resonance imaging (MRI) for prediction of malignant and borderline malignant ovarian tumours. **Methods**: 195 consecutive patients with suspected primary epithelial ovarian cancer were included from the retrospective “Prognostic and Diagnostic Added Value of Medical Imaging in Staging and Treatment Planning of Gynaecological Cancer” (PRODIGYN) study. The radiological stage, according to the International Federation of Gynaecology and Obstetrics system (rFIGO), magnetic resonance imaging (MRI)-based Ovarian-Adnexal Reporting and Data System (O-RADS-MRI) score, and the mean apparent diffusion coefficient (ADC_mean_) were investigated for prediction of ovarian malignancy, with histopathology as reference. The same imaging biomarkers were applied to the borderline tumour cohort (n = 33) to predict malignant/adverse features, such as micro-invasion. **Results**: The rFIGO stage demonstrated high accuracy for ovarian malignancy, with an area under the curve (AUC) of 0.98 (95% confidence interval (CI) = 0.97–0.99). On lesion level, the sensitivity and specificity of the O-RADS-MRI score to predict ovarian malignancy, after adjusting for correlated data structure, was 1 (CI: 0.96–1) and 0.82 (CI: 0.70–0.90), respectively. The performance of ADC_mean_ to predict ovarian malignancy on lesion level was moderately high, with AUC = 0.78 (95% CI 0.68, 0.88). Discrimination of adverse features in borderline tumours was not improved. **Conclusions**: rFIGO and O-RADS-MRI showed excellent performance and outperformed ADC_mean_ as predictive tools for ovarian malignancy but could not predict adverse features in borderline tumours.

## 1. Introduction

Ovarian-adnexal tumours include malignant, benign, and borderline tumours of unknown malignant potential. Ovarian cancer ranks as the seventh most common cancer in women [1]. Histopathological subtypes of primary ovarian cancer can be derived from epithelial cell lines; epithelial ovarian cancer (EOC), which accounts for 90% of cases; or more rarely, from non-epithelial origin, including germ cell tumours and sex chord-stromal tumours [2]. The most common benign ovarian masses are serous cystadenoma (67%), mucinous cystadenoma (19%), and dermoid tumours (11.6%) [3]. In borderline ovarian tumours, serous and mucinous histologic subtypes are the most common (65%) [4], while the remaining rare subtypes consist of seromucinous, endometrioid, clear cell, and Brenner tumours [5]. 

Ovarian cancer has an overall five year survival rate of 38% in Europe [6] and 48% globally [7]. The poor prognosis is mainly related to the late onset of symptoms and diagnosis at an advanced stage. Furthermore, in premenopausal women, the choice of therapy may affect fertility. In borderline tumours, the risk of disease recurrence is related to the presence of so-called adverse features: stages according to the International Federation of Gynaecology and Obstetrics (FIGO) classification, peritoneal implants, microinvasion, and—for serous borderline tumours—micropapillary architecture [8]. Pre-operative radiological characterization and staging of ovarian tumours, and recognition of adverse features in borderline tumours, are crucial for optimized patient management [9].

The 2021 revision of the FIGO staging system is mainly based on surgical findings [10]. Additional radiological staging information (rFIGO) may be included for the determination of the preoperative FIGO stage [10]. Imaging plays a vital role in predicting the likelihood of malignancy, with ultrasound (US), magnetic resonance imaging (MRI), and computed tomography (CT) as primary diagnostic imaging modalities for ovarian-adnexal tumours [11]. Around a quarter of adnexal masses are challenging to characterize with ultrasound [12]. In such cases, MRI, with its high tissue-distinguishing capabilities and specificity in depicting pelvic organs, can be employed to evaluate lesions that are indeterminate or inadequately assessed by ultrasound [13]. Studies have reported an accuracy of 92% sensitivity and 85% specificity in using MRI to differentiate between benign ovarian lesions and borderline/invasive tumours [11].

The standardized Ovarian-Adnexal Reporting and Data System (O-RADS-MRI provides risk stratification of indeterminate adnexal masses and may facilitate correct categorization of borderline ovarian tumours [12]. It can be applied to both US and MRI [14,15]. In comparison with other staging guidelines, all demonstrated high diagnostic performance, and no significant differences were observed in terms of sensitivity or specificity [16]. The O-RADS-MRI score zero is given when the exam is incomplete, e.g., lesion not fully depicted, presence of artefacts, or incomplete MRI sequences. Subsequently, scores 1–5 predict increased probability of malignancy [13,14,17].

The O-RADS-MRI protocol does only briefly include diffusion-weighted imaging (DWI) MRI, which has been shown in previous studies to predict malignancy, especially the quantitative variable mean apparent diffusion coefficient (ADC_mean_) [18]. A study from 2009 [19] showed high accuracy of MRI for detecting benign tumours if a solid mass displayed low signal intensity in high b-value (b1000) DWI MRI, and simultaneously low signal at T2-weighted (T2W) images. A meta-analysis from 2018 [20] showed that the quantitative apparent diffusion coefficient (ADC) values of DWI are useful for differentiating benign from malignant ovarian tumours. ADC may be measured in minimum and mean values; however, there is no difference in the correlation between tumour cellularity and minimum and mean ADC, respectively [21]. The mean ADC value has too much overlap between benign and malignant ovarian lesions for confident use as a diagnostic cut-off parameter [22]. However, for other cancer forms, such as breast cancer, it has been shown that the mean ADC performed better than minimum ADC in the differentiation of benign and malignant lymph nodes [23].

A major limitation of MRI is the dedicated approach, where possible distant metastases are not commonly included in the field of view. The addition of whole-body MRI sequences is an option, but the trade-off between high image resolution and a larger field of view usually results in prioritization of the image quality of the dedicated examination with a smaller field of view. Therefore, for the staging of ovarian cancer in terms of lymph node metastases and distant metastases, contrast-enhanced CT (CECT) is the modality of choice. Peritoneal metastases can be detected with a sensitivity of 92% and specificity of 82%. However, small peritoneal metastases (<1 cm) can be difficult to detect, with a drop in sensitivity to 25–50% [24]. 

A meta-analysis from 2012 [25] shows higher accuracy in the detection of lymph node metastasis with positron emission tomography with combined CT (PET/CT), in comparison to stand-alone CT or MRI. To date, 2fluorine-18-fluoro-deoxy-glucose-(2-[18F]-FDG) is the most used PET radiotracer for metastasis detection in the most common ovarian malignancy EOC and is superior to CT and MRI in detecting non-pathologically enlarged lymph node metastases (<1 cm) [25,26]. Additionally, 2-[18F]-FDG PET/CT can add valuable information in EOC staging [27], especially when it comes to up-staging into the highest stage IV EOC [28]. Due to insufficient evidence regarding the impact of earlier up-staging on survival, 2-[18F]-FDG PET/CT is not recommended for a routine pre-therapeutic check-up in the current version of the Swedish National Guidelines for EOC, but it may have an incremental diagnostic and prognostic value in selected cases [29].

Despite the extensive use of imaging techniques, evidence-based guidelines as well as a recent review article agree on the knowledge gap regarding the optimal imaging modality for primary differentiation of ovarian masses [30].

The main aims of this study were to investigate the diagnostic performance of the imaging biomarkers O-RADS-MRI score, rFIGO stage, and ADC_mean_ for the detection of primary ovarian malignancy, and in a second step, to apply these biomarkers on borderline tumours to identify the presence of adverse features, indicating an increased risk of malignant potential.

## 2. Materials and Methods

### 2.1. Patient Inclusion and Data Retrieval

Data were retrieved from the retrospective part of the PRODIGYN study, Prognostic and Diagnostic Added Value of Medical Imaging in Staging and Treatment Planning of Gynecological Cancer (full prospective study protocol available at Clinical Trials PRODIGYN, NCT05855941, date of registration 23 May 2023), with ethical approval reference number 2022-04207-01. For the retrospective part, informed consent was waived. All consecutive participants in the suspected EOC cohort were included in this study. The data were collected by the Data Extraction team at Region Västerbotten to ensure compliance with the EU general data protection regulation (GDPR). Search criteria were newly diagnosed EOC, no prior treatment, and a case presented at the Region Västerbotten Referral Center Multidisciplinary Tumour Board for Gynecological Oncology during the period of 2016–2022.

Inclusion criteria: high clinical suspicion of primary EOC, not previously treated, known clinical FIGO stage, >18 years old, no other known current or previous malignancy within the last 10 years.

Exclusion criteria: no available MRI scan. Imaging findings suggestive of other primary malignancy.

Additional exclusion criteria for the present study were ovarian torsion, ectopic pregnancy or pelvic inflammatory disease (according to disqualifying criteria for O-RADS-MRI assessment [14]), missing histopathological diagnosis, and borderline tumours with missing information on microinvasion status.

**Figure 1:**
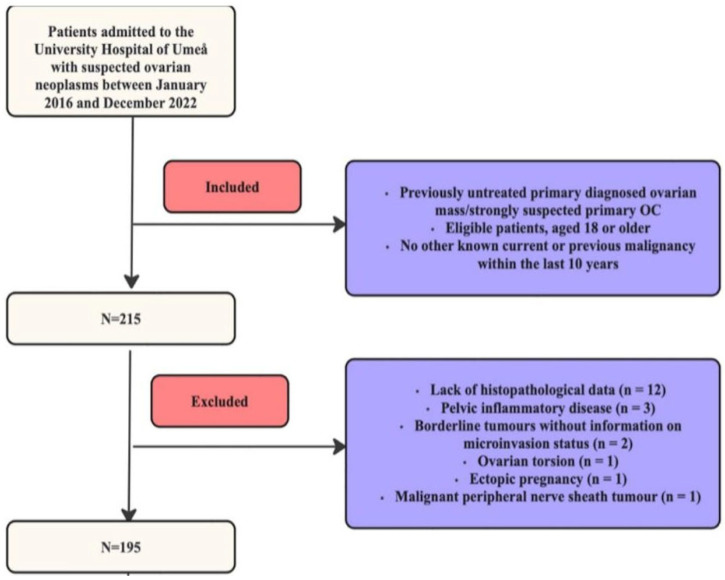
Patient inclusion flowchart.

In total, 215 patients with suspected EOC fulfilled the retrospective PRODIGYN study inclusion criteria. For the present analysis, 20/215 patients were excluded after application of the additional exclusion criteria specified above, leaving 195 patients for inclusion, as illustrated in detail in Figure 1.

Of the 195 included patients, 54 had CA-125 within normal range, <35 U/mL, and 141 had elevated CA-125, mean 1513 U/mL (SD 3100, range 35-19912 U/mL).

### 2.2. Imaging Protocols

167 patients had a pelvic MRI, 193 had a thoraco-abdominal CT and 5 had a whole-body 2-[18F]-FDG PET/CT, all performed prior to treatment. In 2/195 patients, stand-alone CT was missing and instead, the diagnostic CT from the PET/CT was used for the specific CT analyses. Otherwise, the 2-[18F]-FDG PET/CT data were not used in the analysis due to the small number of examinations. All imaging was performed according to clinical routine protocols.

#### 2.2.1. MRI Protocol

MRI examinations were performed on 1,5 Tesla Signa Premier (GE Healthcare, Milwaukee, WI, USA) or 3 Tesla Discovery MR750 scanners (GE Healthcare, Milwaukee, WI, USA). MRI of the pelvis was performed in the supine position using a body array coil following sequences: T2-weighted imaging with and without fat suppression with a slice thickness of 3–4 mm, transverse and sagittal T1 Dixon and/or Wave sequences with a slice thickness of 1 mm, and DWI (using b-values 0, 100, 800/1000)/ADC with a slice thickness of 4 mm. Post-contrast MRI sequences were obtained at baseline and 2.5–3 min after the intravenous administration of gadolinium chelate-based contrast (Dotarem 0.2 mL/kg) at a flow rate of 1–2 mL/s. For post-contrast MRI, transverse and sagittal pre- and post-T1 Dixon and/or Wave sequences were performed with a slice thickness of 1 mm.

#### 2.2.2. CT Protocol

CT examinations were performed on a Siemens Somatom Definition Flash 128-slice scanner (Siemens Healthineers, Forchheim, Germany), GE Lightspeed VCT 128-slice and GE Revolution CT 256-slice CT scanners (GE Healthcare, Milwaukee, WI, USA). Standard thoraco-abdominal CT protocol included oral administration of 1000 mL of water 30 min prior to examination and intravenous bolus injection of low-osmolar contrast medium Omnipaque 350 mg I/mL 0.5 g I/kg up to maximum 80 kg, diluted in 30 mL NaCl. Images were obtained in the supine position from apex to crista in the arterial phase and from diaphragm to trochanter minor in the venous phase with scan parameters of 120 kV (or individualized kV on Siemens Somatom Definition Flash, CARE kV), 80–740 mA, and 0.625 mm slice thickness.

#### 2.2.3. 2-[18F]-FDG PET/CT Protocol

2-[18F]-FDG PET/CT was performed according to clinical routine protocols on a Discovery 690 PET/CT scanner (GE Healthcare, Milwaukee, WI, USA). The CECT was performed after a split-bolus intravenous injection of Omnipaque 350 mg I/mL, 0.5 g I/kg with 120 kV, 150–700 mAs Auto-mA (35 Noise Index), and 0.625 mm slice thickness. All examinations were performed in the supine position with arms above the head, covering a field-of-view from the orbitomeatal plane to the proximal thighs.

### 2.3. Imaging Evaluation

The reading of all imaging examinations was performed independently in a blinded manner by a radiology/nuclear medicine resident (AK). Equivocal cases were classified in consensus with a senior consultant licenced in both Radiology and Nuclear Medicine with >10 years’ experience from gynecological-oncological radiology (SS). The rFIGO stage was determined on patient level, for patients with at least one primary malignant ovarian tumour and for patients with benign tumours only. For all primary malignant, benign, and borderline ovarian masses, O-RADS-MRI score and ADC_mean_ (minimum area 30 mm^2^) were independently assessed. The malignant ovarian tumours of secondary origin were not included in the imaging biomarker analysis.

The histopathology reports were performed in the clinical routine setting, where the pathologists had access to the clinical pre-operative radiological reports.

The same imaging parameters were tested on the borderline tumour cohort to distinguish tumours with adverse features, such as micro-invasion on histopathology.

### 2.4. Statistical Analysis

Receiver operating characteristic (ROC) curves were generated to evaluate the discriminative ability (sensitivity and specificity) of imaging criteria ADC_mean_ and O-RADS-MRI on lesion level, and rFIGO on patient level, to detect primary ovarian malignancy, and to predict borderline tumours with adverse features.

At patient level, ROC curves were constructed for rFIGO staging, and 95% confidence intervals (CIs) for the area under the curve (AUC) were derived from the empirical distribution. At lesion level, ROC curves were generated for ADC_mean_ values. To account for clustering of multiple lesions within patients, we used a patient-level cluster bootstrap with 1000 resamples. For each bootstrap sample, we re-fitted the generalized estimating equations (GEE, exchangeable working correlation structure) model, obtained predicted probabilities, and computed ROC curves and AUC values. Pointwise, 95% CIs for the ROC curves were calculated as the 2.5th and 97.5th percentiles of the bootstrap sensitivity distribution across a grid of specificities. The 95% CI for the AUC was similarly obtained from the bootstrap distribution of AUC estimates.

All statistical analyses were performed with a significance level of <0.05.

There were no substantial missing imaging data, and therefore, no additional statistical handling was required.

## 3. Results

### 3.1. Patient Level

103/195 patients had at least one malignant ovarian tumour, of which 82/103 were primary and 21/103 secondary (metastatic) ovarian tumours. A total of 59/195 patients had only benign ovarian tumours. A total of 33/195 patients had at least one borderline ovarian tumour, of which 8/33 showed adverse features (micro-invasion), and 25/33 did not. A total of 69 patients had bilateral ovarian tumours, of which 59/69 (86%) had the same pathology in both ovaries.

The rFIGO score demonstrated high discriminatory power in distinguishing primary malignant tumours from benign ovarian tumours on patient level, with an area under the ROC curve (AUC) of 0.98 (95% CI = 0.97–0.99).

Frequencies of primary malignant and benign ovarian tumours on patient level for each rFIGO stage are illustrated in Table 1; borderline tumours and malignant ovarian tumours of secondary origin are not included.

**Figure 2:**
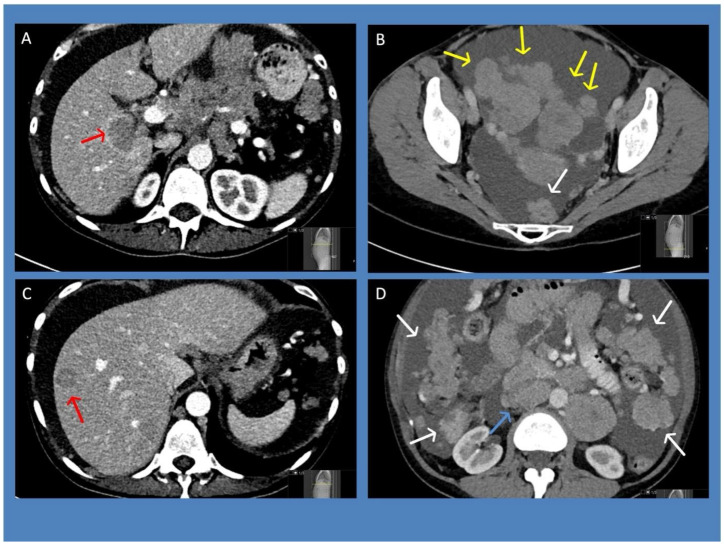
Stage determinant CT findings in a stage IVB EOC. (**A**,**C**) show liver metastases (red arrows). (**B**) shows bilateral primary malignant ovarian tumours (yellow arrows) and a peritoneal implant in the Douglas fossa (white arrow). Figure (**D**) shows bilateral peritoneal and omental implants (white arrows) and a retroperitoneal lymph node metastasis (blue arrow).

The highest rFIGO IV was found in 34/195 patients, all with confirmed primary ovarian malignancy. CT findings in a representative rFIGO stage IVB study participant are presented in Figure 2.

### 3.2. Lesion Level

The frequencies of all malignant, benign, and borderline tumours on lesion level are presented in Table 2.

All primary malignant ovarian tumours were scored O-RADS-MRI 4 or 5; see Table 3 for distribution of primary malignant versus benign and borderline ovarian tumours on lesion level for different O-RADS-MRI scores.

The sensitivity and specificity of O-RADS-MRI to predict primary ovarian malignancy on lesion level (with cut-off value of O-RADS-MRI score 4 and adjusted for correlated data structure) was 1 (CI: 0.96–1) and 0.82 (CI: 0.70–0.90), respectively. O-RADS-MRI 0 lesions were excluded from this analysis.

**Figure 3:**
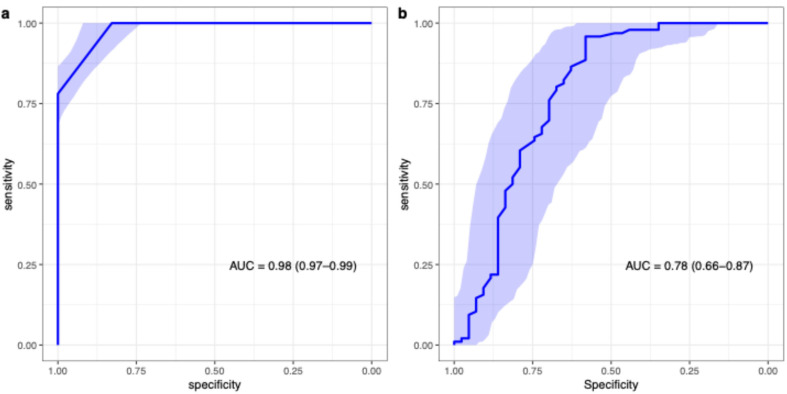
AUC for rFIGO on patient level (**a**), and ADCmean on lesion level (**b**), for prediction of ovarian malignancy.

The mean value of ADC_mean_ was 0.71 (0.61–0.83) 10^−3^ mm^2^/s for primary malignant tumours, and 1.10 (0.76–1.61) 10^−3^ mm^2^/s for benign and borderline tumours. The performance of parameter ADC_mean_ to predict primary ovarian malignancy was AUC = 0.78 (95% CI 0.68, 0.88). Figure 3 demonstrates ROC analysis for rFIGO (a), and ADC_mean_ (b), for prediction of ovarian malignancy.

**Figure 4:**
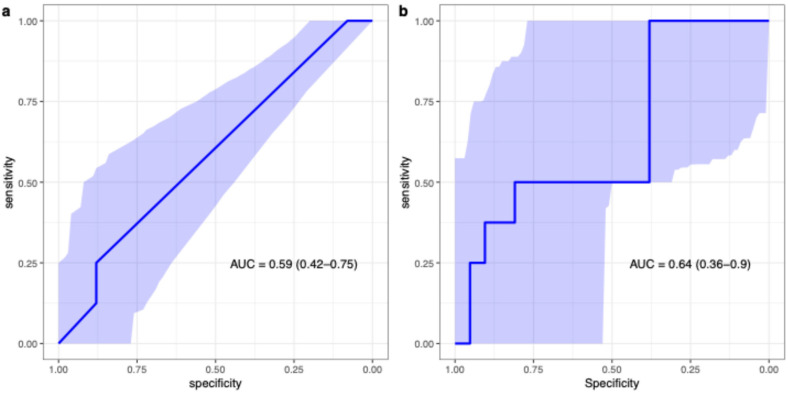
AUC for rFIGO (categorical parameter) on patient level (**a**), and ADCmean (continuous parameter) on lesion level (**b**), for prediction of borderline tumours with adverse features.

None of the rFIGO stage (AUC 0.59), O-RADS-MRI score (sensitivity and specificity 1 (95% CI 0.63–1) and 0.33 (95% CI 0.16–0.55), respectively), or ADC_mean_ (AUC 0.64) showed any obvious tendencies to improve discrimination of adverse features in borderline tumours (ROC analysis illustrated in Figure 4).

## 4. Discussion

Our main results showed that rFIGO stage and O-RADS-MRI score had excellent performance and outperformed ADC_mean_ as predictive tools for primary ovarian malignancy.

### 4.1. rFIGO, O-RADS-MRI and ADC_mean_ as Predictors for Primary Ovarian Malignancy

The fact that the rFIGO stage can be used as a discriminator between benign and primary malignant ovarian conditions is not surprising, given that rFIGO is not used for clearly benign lesions, thereby introducing a bias in the analysis. However, our study illustrates that for lesions of indeterminate malignant potential, rFIGO > I is a reliable indicator for primary ovarian malignancy.

The O-RADS-MRI scoring system is highly effective in pre-operative evaluation of ovarian masses [31]. O-RADS-MRI misclassification of benign lesions from O-RADS-MRI score three to potentially malignant scores of four or five occurs in only 9% [32]. In our study, all primary malignant ovarian tumours were scored O-RADS-MRI four or five, yielding an excellent performance of O-RADS-MRI.

ADC_mean_ did not perform as high as the two compound imaging parameters rFIGO stage and O-RADS-MRI score, but for being a single imaging parameter, the results of ADC_mean_ were impressive, and support the vast amount of previous data on its usefulness in discriminating between malignant (low value) and benign (high value) lesions.

### 4.2. rFIGO, O-RADS-MRI and ADC_mean_ as Predictors for Adverse Features in Borderline Tumours with Increased Malignant Potential

Inclusion of DWI and ADC maps into the assessment of O-RADS-MRI may help reduce the risk of misclassification and may be complementary when the post-contrast time-intensity curve cannot be measured due to hysterectomy or uterine agenesis, with an increase in the sensitivity and specificity of O-RADS-MRI to 84.9% and 95.9%, respectively [33].

DWI and ADC values can be used to differentiate borderline tumours from malignant ovarian tumours, with a higher ADC_mean_ value (1.56–1.77 × 10^−3^ mm^2^/s) in borderline lesions than in malignancies (ADC_mean_ 0.84–1.25 × 10^−3^ mm^2^/s) [34]. We could not reproduce this result on the sub-analysis level when testing for imaging parameters to detect borderline tumours with adverse features suggestive of higher malignant potential. Neither ADC_mean_ nor rFIGO, or O-RADS-MRI showed any obvious tendencies to improve discrimination between microinvasive and non-microinvasive borderline tumours, but the results must be interpreted with care because of the small sample size. Furthermore, it can be questioned if it is reasonable to assume that CT or MRI could detect microinvasion, as these parts of the lesion per definition are smaller than 5 mm in diameter, and also pose a diagnostic challenge for the pathologist. However, radiological detection of other adverse features such as the presence of a micropapillary growth pattern in serous borderline tumours and surface involvement, presenting as irregular and diffuse tumour delineation, is considered feasible.

### 4.3. Bilateral Ovarian Lesions

Bilateral ovarian lesions were of the same entity, to a high extent. In the cases with different diagnoses, concurrent serous and mucinous tumours were found only occasionally. This is consistent with the literature data, which indicate that epithelial tumours of different lineage usually do not occur in both ovaries simultaneously, although several exceptions have been reported confirming the opposite [35,36]. The higher prevalence of identical bilateral adnexal pathology is a clinically relevant finding and may help in the pre-operative reading and reporting of pelvic imaging.

The major strengths of this study concept are the histopathological validation and the structured reading of the CT and MRI examinations. The tested compound parameters rFIGO and O-RADS-MRI are robust and repeatable measures. The singular imaging parameter ADC_mean_ is also considered moderately robust.

The retrospective inclusion process poses an inherent selection bias. However, this material reflects a real-life clinical setting and should be considered as such.

Other limitations of this study concept are the retrospective design, the inclusion bias with only suspected primary EOC, the approach with only one reader, although there were two readers for consensus in selected cases, and the sparse clinical information available due to data protection regulations. The latter particularly hampers generalisability assessment. The sample sizes for subgroup analyses are small, which reduces statistical reliability, and these results must therefore be carefully interpreted.

## 5. Conclusions

rFIGO stage and O-RADS-MRI score showed excellent performance and outperformed ADC_mean_ as predictive tools for primary ovarian malignancy but could not predict adverse features in borderline tumours.

## Figures and Tables

**Table 1:** rFIGO on patient level.

rFIGO Stage	≥1 Primary Malignant Tumour (n = 82)	Benign Tumours Only (n = 64)
0	0 (0%)	53 (83%)
1	18 (22%)	11 (17%)
2	2 (2.4%)	0 (0%)
3	28 (34%)	0 (0%)
4	34 (41%)	0 (0%)

**Table 2:** Distribution and characterization of all included ovarian tumours on lesion level.

**Tumour classification**	**Lesion level (n = 280)**
Malignant	155/280
Benign	85/280
Borderline	40/280
**Malignant subgroups**	**Lesion level (n = 155)**
Primary ovarian malignancy	122/155
Secondary malignancy	33/155
**Borderline subgroups**	**Lesion level (n = 40)**
Borderline with adverse features (micro-invasion)	9/40
Borderline without adverse features (without micro-invasion)	31/40

**Table 3:** Frequencies of primary malignant versus benign and borderline ovarian tumours on lesion level for different O-RADS-MRI scores (2–5 and 0).

O-RADS-MRIscore	Primary Malignant Lesions (n = 122)	Benign Lesions (n = 85)	Borderline Lesions (n = 40)
0	3 (3%)	4 (5%)	3 (10%)
2	0 (0%)	28 (39%)	0 (0%)
3	0 (0%)	28 (39%)	3 (10%)
4	21 (21%)	10 (14%)	19 (63.3%)
5	75 (76%)	2 (3%)	5 (16.7%)
MRI missing	23	13	10

## Data Availability

The de-identified research data in this study will be made available upon reasonable request.
